# Extended Multimodal Flat Detector CT Imaging in Acute Ischemic Stroke: A Pilot Study

**DOI:** 10.1007/s10278-022-00699-4

**Published:** 2023-01-17

**Authors:** Philip Hoelter, Stefan Lang, Vanessa Beuscher, Bernd Kallmuenzer, Michael Manhart, Stefan Schwab, Arnd Doerfler

**Affiliations:** 1 Department of Neuroradiology, University Hospital Erlangen, Friedrich-Alexander-Universität (FAU) Erlangen- Nürnberg, Schwabachanlage 6, 91054 Erlangen, Germany; 2 Department of Neurology, University Hospital Erlangen, Friedrich-Alexander-Universität (FAU) Erlangen-Nürnberg, Schwabachanlage 6, 91054 Erlangen, Germany; 3 Siemens Healthcare GmbH, Advanced Therapies, Siemensstr. 1, 91301 Forchheim, Germany

**Keywords:** Flat Detector Computed Tomography, Interventional, Stroke, Workflow

## Abstract

By using Flat detector computed tomography (FD-CT), a one-stop-shop approach in the diagnostic workup of acute ischemic stroke (AIS) might be achieved. Although information on upstream vessels is warranted, dedicated FD-CT protocols which include the imaging of the cervical vasculature are still lacking. We aimed to prospectively evaluate the implementation of a new multimodal FD-CT protocol including cervical vessel imaging in AIS patients. In total, 16 patients were included in this study. Eight patients with AIS due to large vessel occlusion (LVO) prospectively received a fully multimodal FD-CT imaging, including non-enhanced flat detector computed tomography (NE-FDCT), dynamic perfusion flat detector computed tomography (FD-CTP) and flat detector computed tomography angiography (FD-CTA) including cervical imaging. For comparison of time metrics and image quality, eight AIS patients, which received multimodal CT imaging, were included retrospectively. Although image quality of NE-FDCT and FD-CTA was rated slightly lower than NE-CT and CTA, all FD-CT datasets were of diagnostic quality. Intracerebral hemorrhage exclusion and LVO detection was reliably possible. Median door-to-image time was comparable for the FD-CT group and the control group (CT:30 min, IQR27-58; FD-CT:44.5 min, IQR31-55, *p* = 0.491). Door-to-groin-puncture time (CT:79.5 min, IQR65-90; FD-CT:59.5 min, IQR51-67; *p* = 0.016) and image-to-groin-puncture time (CT:44 min, IQR30-50; FD-CT:14 min, IQR12-18; *p* < 0.001) were significantly shorter, when patients were directly transferred to the angiosuite, where FD-CT took place. Our study indicates that using a new fully multimodal FD-CT approach including imaging of cervical vessels for first-line imaging in AIS patients is feasible and comparable to multimodal CT imaging with substantial potential to streamline the stroke workflow.

## Introduction

Expeditious endovascular treatment (EVT) is the therapy of choice in patients with acute ischemic stroke (AIS) due to large vessel occlusion (LVO) of the anterior circulation [1–4]. Even patients in an extended time window up to 24 h after symptom onset, could benefit from EVT [5]. However, time remains crucial, as it has been shown that the door-to-puncture time and the imaging-to-puncture time, respectively, are independent predictors of successful recanalization and subsequent better outcome [6, 7]. Continuous time surveillance and frequent feedback become more important to extinguish causes of delay and to maintain and improve an effective workflow. Although time from patient arrival in the emergency room (ER) to beginning of EVT has improved from two hours to one hour, transfer remains a crucial bottleneck within a fast AIS workflow [7, 8].

The idea to perform diagnostic and therapeutic imaging within the angiosuite is obvious, as a prolonged time interval from brain imaging to reperfusion negatively effects the outcome [9].

However, the majority of hospitals is not capable to perform EVT and the patients are transferred to an endovascular capable center (ECC) [10]. Due to deterioration and prolonged transportation times, a second multimodal computed tomography (CT) at the ECC might be necessary to decide whether EVT is still indicated. To save time at the ECC, the implementation of a “one stop” approach in the workup of ischemic stroke within the angiosuite is aspired. Studies on “one-stop” management have already been published [11–13]. These studies used flat detector computed tomography (FD-CT), a broadly used imaging technology in interventional radiology and neuroradiology, that offers images with high spatial resolution [14]. However, none of these trials reported on a fully multimodal flat detector computed tomography (FD-CT) approach, that is capable of a dedicated angiographic imaging of the cervical vasculature. Hence, the suggested FD-CT workflow protocols lack of visualization of the cervical vasculature. Thus, important information for EVT planning, e.g., tortuous anatomy, upstream stenosis or dissection, may be missed initially, although extracranial stenosis of the internal cervical artery (ICA) is an important cause of LVO, causing up to 16% of AIS [15].

The purpose of our study was to analyze if fully multimodal FD-CT as an imaging approach is (i) feasible and (ii) expedites the current workflow for outpatients with AIS, when compared to conventional multimodal CT.

## Materials and Methods

### Study Population

Informed consent was obtained by the patients or legal representatives according to local law and regulations. Institutional review board approval was obtained before the commencement of this study and this study was performed in accordance with the ethical standards laid down in the 1964 Declaration of Helsinki and its later amendments.

A total of 16 patients were included. Inclusion criteria were a National Institutes of Health Stroke Scale (NIHSS) > 3, age > 18 years, AIS symptoms due to suspected LVO and transfer from non-endovascular capable centers (nECC) to receive EVT (drip and ship model). To compare time metrics without any bias due to performed general anesthesia (GA), only patients with externally performed GA were included. Moreover, to avoid exposing patients to unnecessary radiation, only patients were included in which an externally performed imaging was not delivered, so new imaging before EVT was required for treatment decision (hemorrhage exclusion, LVO detection, situation of brain hemodynamics).

Our patient collective consisted of two parts. First, we screened prospectively for patients eligible for multimodal FD-CT. Out of 163 AIS patients that received EVT due to LVO between January 2020 and October 2020 (10 months), eight patients met the inclusion criteria and were transferred to the angiosuite, where multimodal FD-CT imaging was performed before EVT due to clinical indication.

Second, we screened our database for AIS patients that received EVT in our department and received multimodal CT imaging before transfer to the angiosuite. Between August 2017 and January 2019 (15 months), 261 patients with LVO received EVT in our department. During that period eight patients, that were screened retrospectively, met the inclusion criteria.

**Fig. 1:**
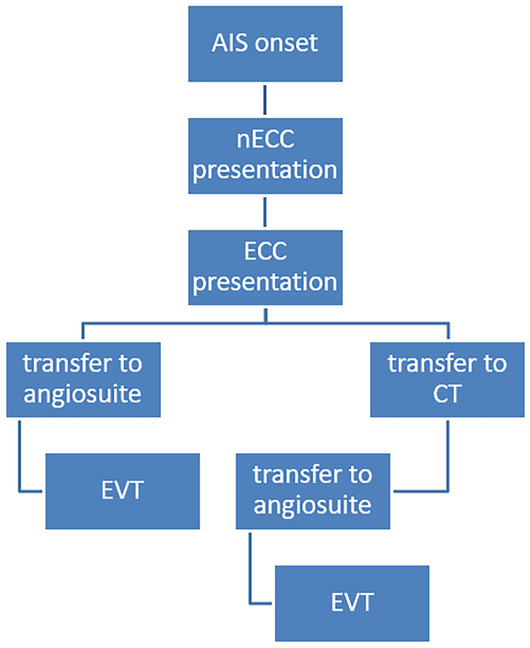
The Workflow that was used. Patients presented externally at an nECC with suspected LVO. GA took place at the nECC or before. Afterwards, the patients were presented at our ECC. In our cohort external performed imaging was not presented to the ECC neuroradiology department. When the interventional neuroradiologist demanded additional imaging, multimodal CT imaging was performed before EVT. After establishing our new FD-CT protocol multimodal imaging was performed in the angiosuite using FD-CT

For all patients major workflow times were recorded: Time of AIS onset; Time of arrival in the ER of our center; Time of cerebral imaging; Time of groin puncture; Time of last DSA series. From the recorded data, several time intervals were calculated: Symptom onset to initial imaging (onset-to-image); ER arrival to initial imaging (door-to-image); ER arrival to groin puncture (door-to-groin-puncture); initial imaging to groin puncture (image-to-groin-puncture); initial imaging to reperfusion (image-to-reperfusion); groin puncture to reperfusion (groin-puncture-to-reperfusion). “Groin puncture” was defined as the moment when the interventionalist cannulates the inguinal artery to gain access to the patient's arterial system, thereby beginning the EVT procedure. “Reperfusion” was defined as the end of EVT, irrespective of the achieved TICI score. Workflow is pictured in Fig. 1.

### Multimodal CT

CT was performed using a 128-section scanner (Somatom Definition AS + ; Siemens Healthcare GmbH, Forchheim, Germany). A multimodal stroke protocol (non-enhanced computed tomography (NECT), CTA and computed tomography perfusion (CTP)) was performed.

CTA was performed in caudocranial direction with a coverage in the z-axis was from the aortic arch to the cranial vertex (120 kV, 160 mAs, collimation: 128 × 0.6 mm, rotation time: 0.3 s). 60 ml contrast agent (Imeron® 400, Bracco Imaging, Konstanz, Germany) were injected via an 18-gauge cubital-vein-cannula at a rate of 5 ml/s, followed by 50 ml of saline flush. Bolus tracking was performed in the ascending aorta and image acquisition started 4 s after contrast exceeding 100 HU [16].

CTP was performed in caudocranial direction. Coverage in the z-axis was 96 mm, centered in the basal ganglia (80 kV, 80 mAs). Acquisition of one scan every 1.5 s over a period of 67.98 s was initiated with a delay of 2 s after contrast injection. 30 ml contrast agent (Imeron® 400, Bracco Imaging, Konstanz, Germany) were injected via an 18-gauge cubital-vein-cannula at a rate of 5 ml/s, followed by 50 ml of saline flush.

### Multimodal FD-CT

Multimodal FD-CT and digital subtraction angiography (DSA) was performed in one session using the same angiography system (Artis icono; Siemens Healthcare GmbH, Forchheim, Germany) followed by DSA. All FD-CT applications used are CE-labeled. Specific FD-CT protocols were obtained in the following order: 1. non-enhanced FD-CT (NE-FDCT); 2. FD-CT perfusion (FD-CTP); 3. FD-CT angiography (FD-CTA) (Table 1). For FD-CTA, image acquisition was started using bolus watching technique.

**Table 1:** Flat detector protocol parameters of the specific protocols used

	**NE-FDCT** **(7 s DynaCT Sine Spin)**	**FD-CTP** **(60 s DynaCT Head Perfusion)**	**FD-CTA** **(4 s Dyna CT Head Care)**
Angulation	220°	200°	800°
° per frame	0.4	0.8	0.8
kV	109	70	70
FOV	49	49	49
Rotations [n]	1	10	1
Duration per rotation	7 s	5 s (+ 1 s pause between the rotations)	4 s
Duration in total	7 s	60 s	4 s
Amount of contrast used	n/a	50 ml	50 ml

*NE-FDCT* Non-enhanced Flat Detector Computed Tomography, *FDCTP* FDCT perfusion, *FDCTA* FDCT angiography

All contrast agent (Imeron® 400, Bracco Imaging, Konstanz, Germany) used was injected via an 18-gauge cubital-vein-cannula at a rate of 5 ml/s, followed by 50 ml of saline flush.

Perfusion volumes were calculated using RAPID CTP and RAPID ANGIO software (iSchema View, Inc., Menlo Park, Ca, USA). Moreover, image reconstruction was performed on a dedicated Siemens workstation and color-coded maps were generated using a dedicated prototype software (Siemens Healthcare GmbH, Forchheim, Germany).

### Image Quality

All non-enhanced, angiographic and perfusion datasets were evaluated by 2 neuroradiologists (7 and 9 years of experience) with respect to image quality. The quality of the datasets was assessed in a consensus reading by using a five-point scaled grading system: 4 = excellent (high contrast, no artefacts); 3 = good (high contrast; minimal artefacts, e.g., due to movement or metallic implants); 2 = compromised (e.g., reduced homogeneity of contrast); 1 = heavily compromised (low contrast); 0 = not diagnostic (no diagnostic information).

Vascular segments were screened with regard to LVO detection: internal cervical artery (ICA) left/right with the proximal and distal segments, middle cerebral artery (MCA) left/right (proximal M1, distal M1, M2-M3), anterior cerebral artery (ACA) left/right, vertebral artery (VA) left/right (V1-V3, V4), basilar artery (BA), posterior cerebral artery (PCA) left/right. Vessel occlusion was described using a 2-point scale for FD-CTA, CTA and subsequent DSA. FD-CTA and CTA datasets were evaluated separately and in random order, blinded to clinical data. DSA datasets were evaluated in a consensus reading.

### Data Analysis

Normal distribution of all data was assessed using Kolmogorov–Smirnov test. Normally distributed data were analyzed using one-way ANOVA. In absence of a normal distribution, Mann–Whitney-U test was performed. For binary data, we applied a one-tailed Fisher Exact probability test. For correlation between occlusion site as rated on FD-CTA, CTA and DSA respectively, Pearson rank correlation coefficient was applied.

Statistical significance was considered for a *p*-value of less than 0.05.

To assess the interrater agreement of LVO detection, the interrater correlation coefficient (ICC) was used. ICC estimates and their 95% confident intervals (CI) were based on a single rater, absolute-agreement, two-way mixed-effects model [17]. Interpretation of average ICC values was as follows: poor ≤ 0.40; fair = 0.40–0.59; good = 0.60–0.74; and excellent = 0.75–1.00.

All statistical analyses were performed using commercially available software (IBM® SPSS® Statistics Version 19, Chicago, IL, USA).

## Results

### Demographic and Clinical Data

The included 16 patients did not differ significantly with regard to their demographic data. The admission status was not significantly different. Most of the patients received i.v. t-PA. Left-sided stroke occurred more often within the FD-CT group, although not with significant difference (*p* = 0.055). The number of performed EVT-passes did not differ significantly.

Processing of perfusion parameters was feasible in all datasets. There was no significant difference between both patient groups with regard to infarct core (*p* = 0.161), tissue at risk (*p* = 0.249) or mismatch volume (*p* = 0.673). Moreover, both groups did not differ in terms of collateral status obtained by perfusion imaging (*p* = 0.893) (Table 2).

**Table 2:** Baseline characteristics; there was no significant difference between the patient groups, indicating a rather homogenous patient collective

		**Multimodal CT** **(n = 8)**	**Multimodal FDCT (n = 8)**	***p* Value**
**Demographic data**				
Gender^a^, female	4 (50)		2 (25)	0.304
Age^b^, years	65.6 ± 10.1		68.8 ± 8.0	0.503
**Admission status**				
Pre-mRS^c^	0 (0–0)		0 (0–2)	0.505
NIHSS^c^	17.5 (12–34)		12.5 (8–16)	0.058
Left hemispheric stroke^a^	3 (37.5)		4 (50)	0.055
Anterior circulation stroke^a^	3 (37.5%)		7 (87.5%)	0.059
Received i.v. lysis^a^	8 (100)		5 (62.5)	0.1
EVT-passes performed^a^	2.5 (1–3)		3 (2–3)	0.382
**Perfusion Details as calculated by RAPID CTP/RAPID ANGIO**				
CBF^b^	9.88 ± 14.29		38 ± 45.58	0.161
Tmax^b^	89.75 ± 53.40		133.88 ± 88.96	0.249
Mismatch Volume^b^	79.88 ± 44.34		95.88 ± 95.02	0.673
HIR^b^	0.47 ± 0.29		0.45 ± 0.31	0.893

*mRS*, Modified Rankin Scale, *NIHSS* National Institutes of Health Stroke Scale, *I.v* Intravenous

^a^*n* (%)

^b^mean ± SD

^c^median (IQR 25th-75th percentile)

### Image Quality and LVO Detection

Image quality was sufficient to detect LVO in all cases and showed no significant difference between the FD-CT and the CT group (*p* = 0.721). Perfusion datasets were sufficient for further analysis too, regardless of the imaging modality (*p* = 0.645). Image quality was lower for NE-FDCT (*p* < 0.001) and FD-CTA datasets (*p* = 0.001) compared to NECT and CTA data (Table 3). However, all NE-FDCT and FD-CTA datasets were of diagnostic quality.

**Table 3:** Quality metrics; image quality of the non-enhanced CT was significantly superior compared to FDCT imaging, whereas there was no significant difference in image quality regarding the perfusion imaging. Although CT-angiography yielded a significant superior image quality, it did not result into a superior LVO-detection

	**Multimodal CT** **(n = 8)**	**Multimodal FDCT (n = 8)**	***p* Value**
**Image Quality**^a^			
Non-enhanced	4 (4–4)	2.5 (2–3)	** < 0.001**
Perfusion data	4 (3–4)	4 (3–4)	0.645
Angiographic data	4 (4–4)	3 (3–4)	**0.01**
LVO-detection	4 (4–4)	4 (4–4)	0.721

^a^median (IQR 25th-75th percentile)

A total of 304 vessel segments (152 by FD-CTA, 152 by CTA) were scored to define site of LVO. Detailed occlusion sites are listed in Table 4. Correlation between DSA series and rated FD-CTA, CTA, respectively, was excellent for both readers (Reader 1: r = 0.967, *p* < 0.001; Reader 2: r = 0.969, *p* < 0.001). Interrater agreement was excellent for both FD-CTA (ICC = 0.986 (0.929–0.997)) and CTA assessment (ICC = 0.995 (0.976–0.999)).

**Table 4:** Occlusion sites; detailed site of each vessel occlusion as detected by FD-CT and CT was assessed for Reader 1 and 2 separately. Site of occlusion assessed on the DSA images in consensus was considered gold standard

	**Reader 1**		**Reader 2**		
Vascular segment, n (%)	**FD-CT (n = 8)**	**CT (n = 8)**	**FD-CT (n = 8)**	**CT (n = 8)**	**DSA (n = 16)**
Proximal ICA	1 (12.5)	0	1 (12.5)	0	1 (6.3)
Distal ICA	0	1 (12.5)	1 (12.5)	1 (12.5)	2 (12.5)
Proximal M1	4 (50)	1 (12.5)	4 (50)	1 (12.5)	5 (31.2)
Distal M1	1 (12.5)	0	0	0	0
M2/3	1 (12.5)	1 (12.5)	1 (12.5)	1 (12.5)	2 (12.5)
ACA	0	0	0	0	0
VA	0	1 (12.5)	0	2 (25)	2 (12.5)
BA	1 (12.5)	4 (50)	1 (12.5)	3 (37.5)	4 (25)
PCA	0	0	0	0	0

*ICA*, Internal Cervical Artery, *M1* M1 segment of the middle cerebral artery, M2/3 M2/3 segment of the middle cerebral artery, *ACA* Anterior Cerebral Artery, *VA *Vertebral Artery, *BA* Basilar Artery, *PCA* Posterior Cerebral Artery

### Workflow Data

4/16 patients had unknown onset of AIS (*p* = 0.715). Onset-to-image time was without significant difference between both groups (CT: 231 min, IQR 96–367; FD-CT: 203 min, IQR 191–262; *p* = 0.928). 2/8 (25%) patients of the FD-CT group and 3/8 patients (37.5%) of the CT group were presented during off-hours (6 pm till 8 am or on weekend; *p* = 0.721).

Door-to-image time showed no significant difference between the CT group (30 min, IQR 27–58) and the FD-CT group (44.5 min, IQR 31–55; *p* = 0.491).

Image-to-groin-puncture time was significantly shorter for patients that received multimodal FD-CT prior to EVT (14 min, IQR 12–18), when compared to the CT group (44 min, IQR 30–50, *p* < 0.001). Thus, door-to-groin-puncture time was significantly reduced when multimodal FD-CT was applied (CT: 79.5 min, IQR 65–90; FD-CT: 59.5 min, IQR 51–67; *p* = 0.016).

Image-to-reperfusion time (CT: 103 min, IQR 68–119; FD-CT: 99.5 min, IQR 54–129; *p* = 0.875) and groin-puncture-to-reperfusion time (CT: 54 min, IQR 32–75; FD-CT: 88 min, IQR 42–114; *p* = 0.165) showed no significant difference between both groups (Table 5).

**Table 5:** Process times; time metrics that represents patient delivery to the imaging modalities (onset-to-image and door-to-image time) did not differ significantly. Neither did the time metrics that represents the time of patient treatment with EVT (image-to-reperfusion and groin-puncture-to-reperfusion time). The time intervals that include patient arrival, patient imaging, respectively, to the start of the EVT is significantly reduced in the FD-CT group

	**Multimodal CT (n = 8)**	**Multimodal FD-CT (n = 8)**	***p* Value**
**Time interval [min]**^a^			
onset-to-image	231 (96–367)	203 (191–262)	0.928
door-to-image	30 (27–58)	44.5 (31–55)	0.491
door-to-groin-puncture	79.5 (65–90)	59.5 (51–67)	**0.016**
image-to-groin-puncture	44 (30–50)	14 (12–18)	** < 0.001**
image-to-reperfusion	103 (68–119)	99.5 (54–129)	0.875
groin-puncture-to-reperfusion	54 (32–75)	88 (42–114)	0.165

^a^median (IQR 25th-75th percentile)

## Discussion

**Fig. 2:**
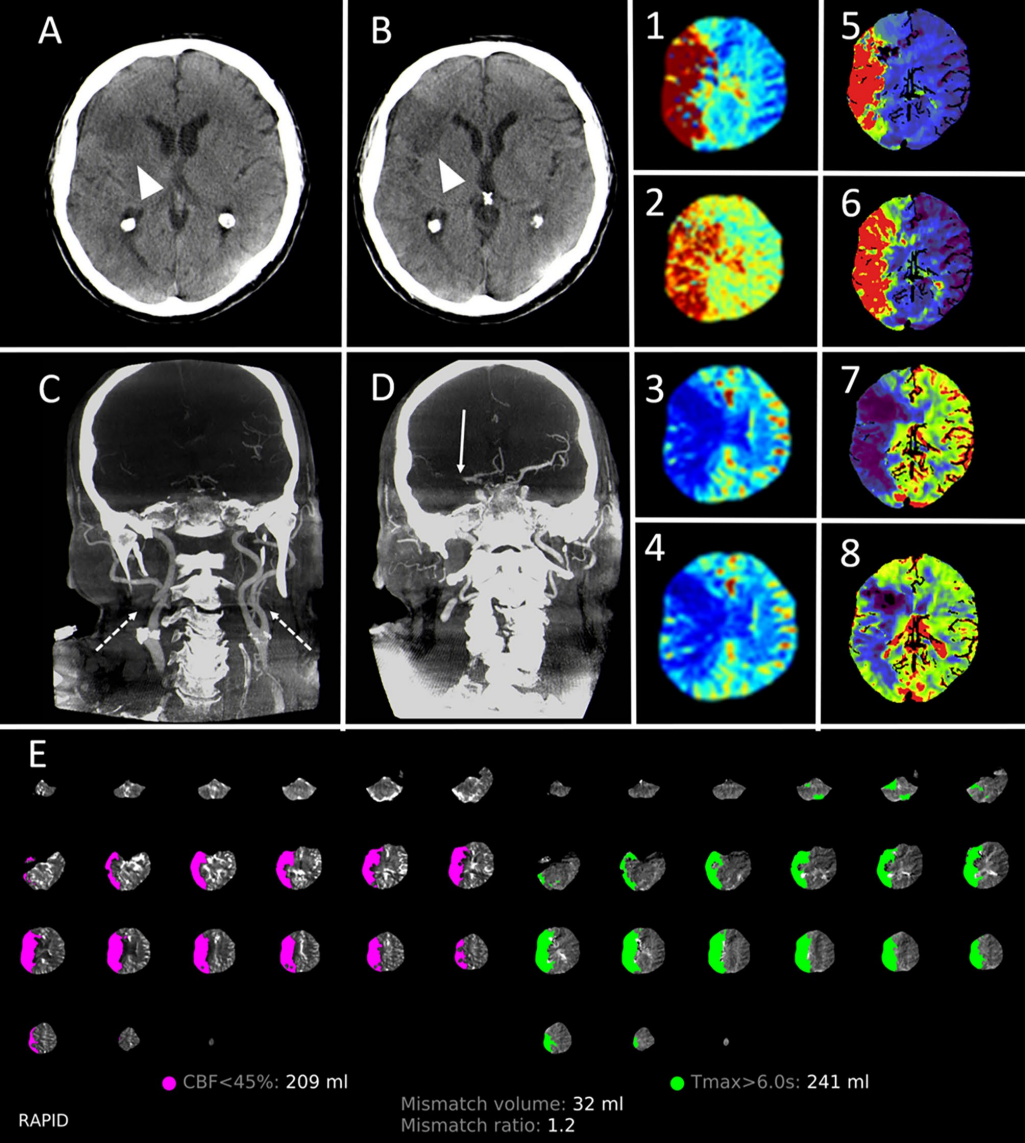
Exemplary images processed by the new multimodal FD-CT approach. 66-year-old male with a proximal M1 occlusion right, NIHSS 21. NE-FDCT on which the demarked infarct of the anterior third of the right MCA-territory can be delineated (**A** and **B**; arrowheads). FD-CTA shows a clear visualization of the cervical vasculature (**C**; dashed arrows) and a proximal occlusion of the right M1-segment (**D**; continuous arrow). FD-CTP shows a matching deficit in the color-coded maps of RAPID ANGIO (1–4) and of the Siemens perfusion prototype software (5–8) as they show a delay for Tmax (1) and TTP (5), MTT (2 and 6) and a reduction for CBF (3 and 7) and CBV (4 and 8). Automated perfusion analysis using RAPID ANGIO revealed a small mismatch between the infarct core, estimated by the CBF reduction (pink), and the potential salvageable tissue, estimated by the delayed Tmax (green), which results into a mismatch ratio of 1.2 (E)

**Fig. 3:**
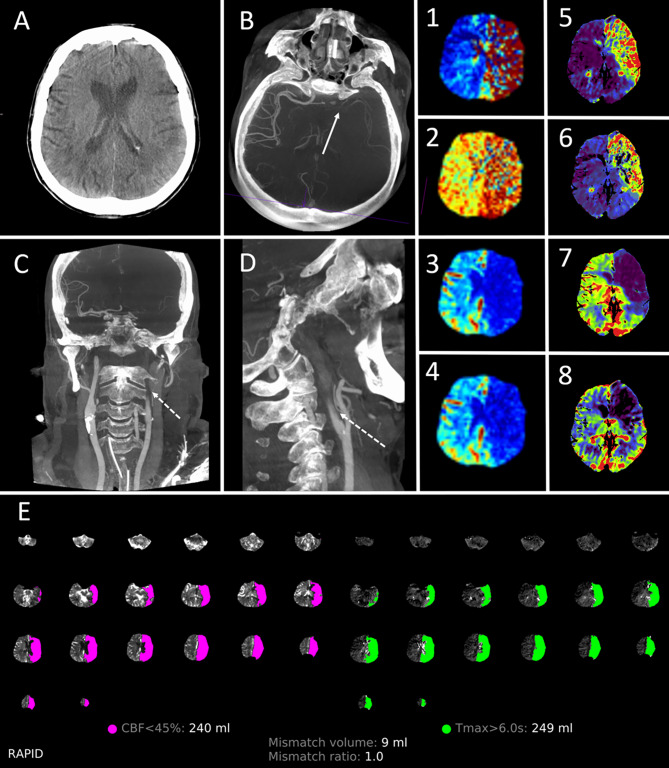
Exemplary images processed by the new multimodal FD-CT approach. 79-year-old female with an occlusion of the Carotid-T left, NIHSS 16. NE-FDCT showed no demarked infarct (**A**). FD-CTA reveals absent filling of the left MCA-territory with almost no collateralization (**B;** continuous arrow) due to an occlusion of the proximal ICA on the left (**C** and **D**; dashed arrows). Color-coded maps of RAPID ANGIO (1–4) and of the Siemens perfusion prototype software (5–8) show a severe prolongation for Tmax (1) and TTP (5), MTT (2 and 6) and a significantly reduced CBF (3 and 7) and CBV (4 and 8). Brain hemodynamics were severely impaired, as the automated perfusion analysis using RAPID ANGIO showed a poor mismatch ratio of 1.0 (E). The ratio was calculated by the division of the potential salvageable tissue, estimated by the delayed Tmax (green), and the presumed infarcted brain, represented by the CBF reduction (pink)

In this small series, we could show that using a new fully multimodal FD-CT approach for diagnosis of LVO in AIS is feasible. Moreover, in our series door-to-groin-puncture times were reduced significantly compared to conventional multimodal CT even though NE-FDCT, FD-CTP and FD-CTA is performed. To our knowledge, this is the first study examining this new FD-CT application in outpatients with AIS due to LVO (Figs. 2 and 3).

Current work of Psychogios et al*.*, who investigated the impact of first-line FD-CT imaging on workflow in AIS patients who presented at the ECC first, draw comparable conclusions as we did in our study: first-line imaging within the angiosuite can significantly reduce intrahospital delay [11].

In line with other current literature our study indicates that by using FD-CT first-line, ‘one-stop-shopping’ can be achieved in outpatients, which reduces the door-to-groin-puncture time and consecutively door-to-reperfusion time [18, 19]. Especially in a clinical setting, with long distances between ER, CT and the angiosuite, time delay by intrahospital transit plays a large role. As Khatri et al. demonstrated, a 30-min delay in angiographic perfusion reduces the relative likelihood of good clinical outcome (mRS of 0–2) by 14% [20]. FD-CT could offer the opportunity to reduce this delay as the transit concentrates on the direction from the ER to the angiosuite.

However, when compared to aforesaid studies there are methodical differences to our new FD-CT approach. As 15% of all AIS are based on carotid tandem occlusions, defined as both, extra- and intracranial occlusion, the need of imaging cervical vasculature is obvious [20]. Previous multimodal FD-CT imaging trials lack on imaging cervical vasculature [13]. Our approach aims for a fast imaging of the cervical vasculature within 4 s of scanning time using bolus watching technique. The study group of Psychogios et al*.* used a biphasic angiographic approach [11, 13]. This technique offers high-quality images of the intracranial vasculature, however, limited to the skull [21]. With our new multimodal FD-CT protocol including a FD-CTA acquisition with extended FOV imaging from the aortic arch up to M3 segment level is feasible instead. Despite FD-CTA offers information on cervical vasculature, it is limited by its FOV, which currently covers 49 cm. Thus, imaging from aortic arch and full brain coverage is not feasible. Potential protocol adjustments, e.g., FD-CTA for visualization of the cervical vasculature and FD-CTP for visualization of the cerebral vessel, might be considered to overcome this drawback.

The aforesaid studies did not apply perfusion imaging in their study, whereas our approach includes a dynamic perfusion scan. Subsequently, dedicated high-quality imaging of collateral status and clot burden is possible as previously shown [23]. Recent work of Brehm et al*.* showed reduced door-to-groin-puncture times in AIS patients when applying a perfusion based FD-CT-approach [12]. They used a perfusion protocol, which is similar to that applied in our study. However, as their workflow did not include FD-CTA, imaging of the cervical vasculature was not performed.

To our knowledge, we provide data on real multimodal FD-CT imaging in AIS patients for the first time, proving that additional imaging of the cervical vasculature is feasible within the angiosuite. Thus, a CT-like diagnostic workup of AIS seems possible when using FD-CT only.

In our study, the suggested door-to-groin-puncture-time of less than 60 min was achieved within the FD-CT cohort and is lower as times reported by the HERMES collaboration [24, 25].

Unfortunately, the fast-imaging workup of the FD-CT cohort could not be transferred to faster image-to-reperfusion times (*p* = 0.875). As both groups did not significantly differ with regard to the number of performed EVT passes (*p* = 0.382), this might be addressed by difficult access to the clot, which cannot be affected by the choice of imaging. Moreover, our small and selected study population might have hindered advantageous speed of reperfusion for the FD-CT group. However, our inclusion criterion of the patient cohort was the externally performed GA. This was done to minimize any bias of intrahospital workflow times. The predominance of posterior LVO in the CT group might be partially explained by this approach, as imbalances regarding the site of LVO, or LVO access were not addressed equally. Thus, assessments of larger cohorts of the anterior and posterior circulation are warranted.

In analogy to multimodal CT, reconstructing non-enhanced images, cerebral perfusion images and the cerebral vasculature out of the obtained FD-CT datasets is mandatory. The speed of the image acquisition is short. The acquisition time is 7 s for the NE-FDCT, 60 s for the FD-CTP and another 4 s for the FD-CTA. Reconstruction time is comparable to that of multimodal CT but depends on the operators’ experience. To our knowledge time of post-processing does not play a role when comparing both modalities. In all patients, software reconstruction was successful and offered information of impaired brain hemodynamics.

FD-CT image quality of brain parenchyma is inferior when compared to CT. Especially with regard to the assessment of early ischemic changes and evaluation of the Alberta Stroke Program Early CT Score (ASPECTS), respectively [26]. The delineation of SAH depiction, especially near the skull base, was reported to be reduced as well [27, 28]. However, Leyhe et al*.* recently showed that the latest type of FD-CT detector offers high sensitivity and specificity in intracranial hemorrhage detection [29]. Nevertheless, NE-CT and CTA data were superior in the qualitative rating. The use of an alternative NE-FDCT protocol might overcome this drawback in parts, as there are studies reporting on superior depiction of cerebral vasculature when applying FD-CTA [30]. However, these studies use different programs, dedicated for intracranial vessel imaging. Nonetheless, although image quality was rated inferior for FD-CTA in our study, all FD-CT datasets were sufficient for LVO-detection, just like the CT datasets. Both showed excellent interrater agreement, as well as an excellent correlation to the subsequent DSA series.

To our knowledge this is the first paper describing an “one-stop-shopping” approach with integrated AI-based perfusion assessment performed with RAPID ANGIO. Automated image processing using RAPID software was feasible in all cases. The FD-CTP datasets showed equivalent image quality when reconstructed, offering the information needed to estimate infarct core and penumbra. Especially for readers with less experience in perfusion imaging automated software applications offer support in image evaluation. Whether automated perfusion analysis using RAPID software differs with regard to volumes when comparing CTP and FD-CTP has not been addressed yet. As our cohorts are different with regard to occlusion site—though not significant due to the small number of patients—comparative statements are not possible. Niu et al. showed, that automated FD-CTP postprocessing is comparable to CTP [31]. By using automated software solutions, reconstruction and interpretation of perfusion data might help physicians with clinical decision making. Thus, the workflow might get more streamlined.

With regard to dosage measurement, a comparable amount of doses has already been described for FD-CT and CT imaging [32]. Dedicated measurement of effective doses for the 7 s NE-FDCT Sine Spin run and the 4 s FD-CTA are still missing and are subject of running studies. However, due to the rotation angle of 200° dosimetry on FD-CT in comparison with CT has to be considered as an approximation.

The present study has several limitations. First, it is in parts retrospectively designed and monocentric, which bears the risk of selection bias. Second, our study cohort is relatively small and limited on outpatients that have already received GA. In order to focus on transfer times that are not influenced by GA on site, we decided to choose this inclusion criterion. However, this criterion has hindered this study to include more patients, especially for the retrospective CT cohort. Third, the patients that received CT imaging had predominantly occlusions of the posterior circulation.

## Conclusion

Using a new extended multimodal FD-CT protocol, diagnostic imaging within the angiosuite is a promising attempt to streamline the AIS workflow. Our small series showed that by using FD-CT, the time span between imaging and EVT can be speed-up. However, dedicated workflow-analyses on larger patient cohorts are needed and improvements in organization and interdisciplinary collaboration are essential to guarantee a treatment in shortest time possible.

## Data Availability

The data presented in this study are available on request from the corresponding author. The data are not publicly available due to data protection laws.
